# Prematurity and Low Birth Weight Among Food-Secure and Food-Insecure Households: A Comparative Study in Surabaya, Indonesia

**DOI:** 10.3390/nu17152479

**Published:** 2025-07-29

**Authors:** Arie Dwi Alristina, Nour Mahrouseh, Anggi Septia Irawan, Rizky Dzariyani Laili, Alexandra Vivien Zimonyi-Bakó, Helga Judit Feith

**Affiliations:** 1 Health Sciences Division, Doctoral College, Semmelweis University, 1085 Budapest, Hungary; alristina.arie@phd.semmelweis.hu; 2 Nutrition Department, Sekolah Tinggi Ilmu Kesehatan Hang Tuah Surabaya, Surabaya 60244, Indonesia; rizkylaili@stikeshangtuah-sby.ac.id; 3 Department of Public Health and Epidemiology, Faculty of Medicine, University of Debrecen, 4028 Debrecen, Hungary; nour.mahrouseh@med.unideb.hu; 4 Institute of Behavioral Sciences, Faculty of Medicine, Semmelweis University, 1089 Budapest, Hungary; irawan.anggi@phd.semmelweis.hu; 5 Institute of Languages for Specific Purposes, Semmelweis University, 1091 Budapest, Hungary; bako.alexandra@semmelweis.hu; 6 Department of Social Sciences, Faculty of Health Sciences, Semmelweis University, 1085 Budapest, Hungary

**Keywords:** prematurity, low birth weight, food security, maternal education, child nutrition

## Abstract

**Background**: Prematurity and low birth weight (LBW) drive infant morbidity and mortality, requiring nutritional interventions, especially in food-insecure settings. In Indonesia, regional disparities in food security hinder adequate nutrition for premature and LBW infants, exacerbating health challenges. The aim of study is to investigate and determine factors associated with prematurity and LBW in children from food-insecure and food-secure households. **Methods**: This research employed a cross-sectional study with 657 mothers of children aged 36–59 months, conducted using random sampling. Data was collected via standardized questionnaires and analyzed using Chi-square tests and logistic regression. **Results**: The adjusted model showed that children of food-insecure households had a higher risk of LBW (AOR = 0.54; 95% CI: 0.29–0.99; *p* < 0.05). LBWs were found to significantly less occur in food-insecure households. Low maternal education was associated with an increased risk of preterm birth (AOR = 3.23; 95% CI:1.78–5.84; *p* < 0.001). Furthermore, prematurity correlated with house ownership (*p* < 0.01), indicating the household’s wealth condition. Maternal education and house ownership were linked to prematurity, indicating the risk to child health outcomes. In summary, maternal education, employment status, and household income were linked to food insecurity, indicating the risk to child health outcomes. **Conclusion**: Strategies to improve child health outcomes are essential, including enhancing maternal nutrition knowledge to improve child feeding practices, promoting gender equality in career development, and reducing food insecurity in households.

## 1. Introduction

Globally, neonatal and under-five mortality remains a critical health issue, especially regarding regional health inequalities. Approximately 13.4 million preterm births worldwide were recorded in 2020, leading to an estimated 900,000 deaths in 2019 [1]. Indonesia was in the top five countries for the prevalence of preterm births, recording 675,700 cases in 2018 [2]. In 2023, the national premature birth rate was estimated at 11.1%, with notable regional variations, such as a 6.9% preterm birth rate in East Java [3]. Despite significant progress in reducing prematurity, as well as its impact on neonatal mortality, with significant progress in decreasing cases from 23 per 1000 live births in 2012 to 10.7 per 1000 in 2022 [4], challenges persist. This country has achieved the Sustainable Development Goal (SDG) target of reducing neonatal mortality to fewer than 12 deaths per 1000 live births in each country [5]. Furthermore, prematurity and low birth weight were the leading causes of the 27.9% neonatal mortality rate in East Java in 2023 [6]. Concurrently, urban areas like Surabaya indicated disproportionately high infant mortality rates, highlighting persistent inequalities in healthcare access and maternal health outcomes.

Low birth weight (LBW) is a significant contributor to neonatal mortality, accounting for 60–80% of neonatal deaths each year worldwide, with more than 20 million infants born with LBW annually [7]. LBW, often resulting from preterm birth, is a birth weight of less than 2500 g [8]. LBW may occur in both preterm and full-term birth due to growth retardation and is a major factor in neonatal mortality [9]. According to the World Health Organization (WHO), approximately 15% of babies worldwide experience LBW, with more than half of these cases occurring in Asia [8]. In Indonesia, the prevalence of LBW is recorded at 6.1% [10]. Previous research indicates that LBW is correlated with various factors, such as maternal education [11], socioeconomic status [12], nutritional deficiencies [13], anemia [13], and inadequate antenatal care [5,13,14]. In Indonesia, other factors associated with LBW include family size, nutritional status, maternal age, pregnancy complications, age at marriage, iron supplementation, and anemia status. Additionally, LBW also has a relationship with delayed breastfeeding, feeding refusal, impaired growth, and long-term developmental challenges [15].

The COVID-19 pandemic impacted household food security, inciting a concerning global trend. The Food and Agriculture Organization (FAO) reported that around 828 million people faced hunger in 2021 [16]. Approximately 29.6% of the global population identified moderate to severe food insecurity by the end of 2022 [17]. The prevalence of food insecurity decreased from 35% in 2008 to 20.8% in 2015, which was attributed to rising household incomes and rapid economic growth in Indonesia. However, one in five Indonesians still faces severe food insecurity [18]. A significant finding from the National Socioeconomic Survey in Indonesia in 2021 was that 76% of children under five lived in food-insecure households. The breakdown of food insecurity levels showed that 17% experienced mild, 5% moderate, and 2% severe food insecurity [17]. This context underscores the urgent need for targeted interventions to address food insecurity, as it is closely linked to adverse health outcomes, including malnutrition among children [16].

Prior findings have revealed a significant correlation between food insecurity and adverse birth outcomes, such as prematurity and LBW. Studies indicated that food-insecure households are more susceptible to having infants with LBW and stunting, hence increasing the risk of chronic health problems, neurodevelopmental disorders, and higher mortality rates [19,20,21]. LBW infants face significantly higher mortality risks and long-term health complications, including cardiovascular diseases and cognitive impairments [20,22], with prevalence impacted by maternal factors, environmental factors, and socioeconomic status [23,24]. Food insecurity is complex and dynamic, as it can trigger malnutrition, hunger, growth retardation, overconsumption, and obesity, which can be linked to the double-burden of malnutrition [25]. It is essential to obtain significant information regarding the incidence of low birth weight and its associated factors. This data will help in the formulation of effective interventions.

This study aims to examine these effects in food-secure and food-insecure households to explore further potential health inequalities. Despite these acknowledged risks, research is limited regarding how household food insecurity influences the impact of prematurity and LBW among children aged 36–59 months. Identifying these inequalities is important for developing specific approaches, as food-insecure households may endure more severe consequences, contributing to further long-term health inequalities.

## 2. Materials and Methods

### 2.1. Survey Design

The study was conducted in Surabaya, Indonesia, focusing on children aged 36–59 months and their respective households. A cross-sectional study was carried out across five districts of the city—east, west, center, north, and south. The sampling process followed a multi-stage cluster random sampling approach, utilizing administrative divisions to ensure systematic selection. Initially, two sub-districts were randomly selected from each of the five regions, resulting in a total of ten sub-districts. Within each selected district, respondents were recruited from public health centers participating in a community-based health program in Indonesia (Posyandu) that plays a crucial role in providing basic health services, particularly for mothers and children. The source population for this research comprised all mothers or primary caregivers of children within the specified age range who were attending Posyandu in Surabaya. This approach ensured a geographically representative sample, allowing for a comprehensive analysis of the study variables across different geographic areas. The study was conducted in April 2024. Twelve interviewers were involved in data collection, all of whom were college students in a Nutrition Program.

Previous research recommended statistical methods that allow readers to verify results using the original data [26,27]. This principle should also apply to the description of sample size calculation or power analysis. Consequently, the subsequent considerations must be delineated when determining sample size or statistical power.

GPower 3.1.9.4, a software used to calculate statistical power for various statistical tests, such as *t*-tests, F-tests, χ^2^-tests, z-tests, correlation tests, and other statistical analyses, was employed in this study. GPower can also be used to compute effect sizes and display the results graphically, making it suitable for conducting simulation studies and teaching processes. Cohen suggested that the effect sizes for correlations are 0.1 for “small,” 0.3 for “medium,” and 0.5 for “large” [26]. The author calculates the minimum sample for statistical tests in this research by setting the significance level (α) at 0.05, with an expected statistical power of 95%, and a medium effect size of 0.3. GPower software calculated a minimum sample size of 134 mothers. A study should conventionally meet a statistical power of 0.8 [28]. This study tested statistical power by considering a logistic regression test, two tail tests, and sample size of 657 samples, which showed a result of 0.86.

A total of 657 mothers with at least one child aged between 36 and 59 months were randomly sampled from the public health center’s data. In cases where more than one child met this criterion, only one was selected. The lists of mothers with children under five were obtained four weeks prior to data collection in each of the ten sub-districts. However, children with a diagnosed chronic illness, who were bedridden, or who had suffered a serious acute illness were excluded from the study. The same applied to any child with a physical deformity affecting the lower extremities or spine. Women who were permanent residents or had spent the previous night in the designated households were qualified for interviews.

### 2.2. Data Collection

Participation in this study was voluntary, and mothers were informed about the aim of the research before enrolment. Each participants had to sign an informed consent sheet before completing the questionnaire, as confidentiality and their right to withdraw the participation at any time were ensured. Data was collected using a structured questionnaire and administered face-to-face.

The study examined socioeconomic factors (income, food insecurity) and maternal characteristics (age, education, employment). The analysis followed four steps: (a) descriptive analysis to evaluate relationships between variables; (b) variable selection prioritizing food insecurity, preterm birth, and LBW; (c) multivariable modeling to refine predictors; and (d) finalizing a model including key maternal factors.

Household food insecurity was measured using the Food Insecurity Experience Scale Survey Module (FIES-SM) from the Food and Agriculture Organization of the United Nations with two classifications: food-secure and food-insecure households. The FIES-SM measures food insecurity in the household by applying the Rasch model, determining the range of food insecurity as mild, moderate, or severe [29].

A pilot test was conducted to ensure the reliability and clarity of the questionnaire that underwent pretesting on 5% of the sample. Weighing scales were calibrated daily using a standard weight (1000 g standard reference weight). Enumerators received training in survey administration, response recording, and anthropometric measurements. Daily reviews by the primary investigator ensured accuracy and completeness, with feedback provided as needed.

### 2.3. Statistical Analysis

Data was recorded in Microsoft Excel and analyzed using RStudio Version 2025.05.1+513. Descriptive statistics described socio-demographic and other data points, whereas bivariate analysis was conducted with the Chi-square test and Fisher’s exact test. Logistic regression identified connections among preterm birth, LBW, and household food security and related variables. Variables with *p*-values of less than 0.05 in bivariate analysis and epidemiological interest were incorporated into multivariable regression to control for confounders. Adjusted odds ratios (AOR), accompanied by 95% confidence intervals, evaluated the strength of connections, with *p*-values < 0.05 deemed statistically significant.

## 3. Results

### 3.1. Study Population

A total of 657 mothers with children aged 36–59 months were randomly selected to participate in the study and included in the analysis. Among these, 38.51% of mothers were categorized as food-insecure, while 61.49% were classified as food-secure. Children’s prematurity prevalence was noted at 44.29%, and LBW was reported at 9.28%.

**Figure 1:**
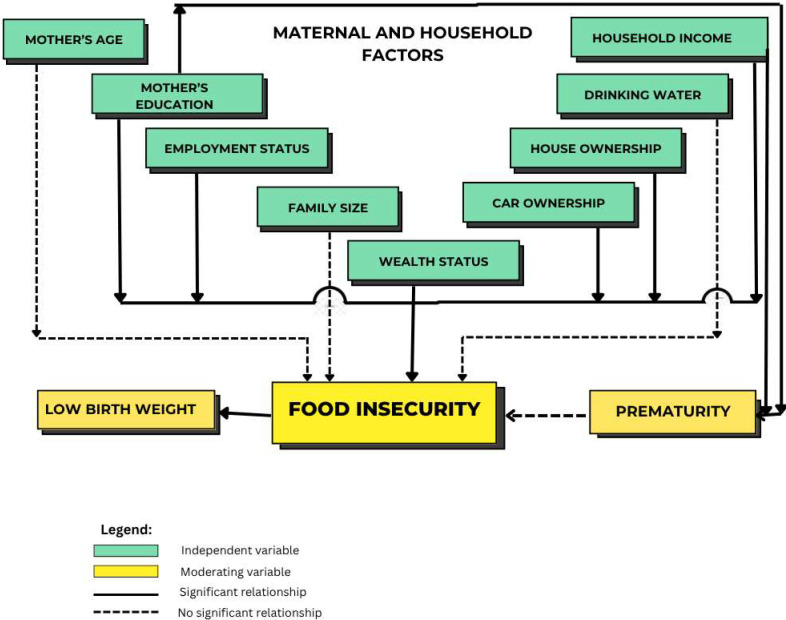
Food insecurity pathway on the impact of prematurity and low birth weight (LBW).

We applied the Chi-square test to examine the relationship between two categorical groups—food-secure households and food-insecure households—and other determinants. Household food insecurity was significantly associated with maternal education (*p* < 0.001) and employment status (*p* < 0.01), as determined by the test. Additional factors influencing food insecurity included monthly household income (*p* < 0.001), car ownership (*p* < 0.001), house ownership (*p* < 0.01), and overall wealth status (*p* < 0.001), as shown in Table 1 and Figure 1. Households with limited access to safe drinking water are more likely to be food-insecure, as only 28.16% consume mineral water and 0.46% consume spring water (Table 1).

As shown in Table 2, preterm birth occurred in 27.85% of food-secure households and 16.44% of food-insecurity households. Most mothers were mature adults aged 30–39, with 23.40% delivering premature children. Maternal education was significantly associated with preterm birth (*p* = 0.008). The highest rate of preterm birth occurred among mothers with medium education, 24.35%. A total of 25.72% of mothers who delivered premature children were not employed. The majority of mothers, 35.77%, were concentrated in the low-income group. Mineral water households were most common among both groups, with 33.03% experiencing a preterm birth and 43.23% a normal birth. There was a significant association between house ownership and preterm birth (*p* = 0.003), with an increase in preterm birth among those without home ownership, at 29.98%. The highest rate of preterm births, 38.05%, occurred in households without a car. Most preterm births occurred among the average wealth group (30.29%).

LBW children accounted for 6.70% in food-secure and 2.59% in food-insecure households. Similarly, maternal age was not significantly associated with LBW, and it was distributed evenly across age groups. Although descriptively the lowest LBW rate was observed among mothers with high education (1.2%), followed by medium (4.4%), and low (3.7%) education, the association was not statistically significant. Employed mothers had a slightly higher LBW rate (4.9%) than unemployed mothers (4.4%). No significant difference in LBW prevalence by family size was found, and most LBW rates (6.70%) were found in medium-sized households. LBW rates were highest among families using mineral water (6.85%). There was no association between car or house ownership. The LBW rates were concentrated in the average wealth status group (6.24%) (Table 1).

### 3.2. Factors Associated with Prematurity, Low Birth Weight, and Household Food Insecurity

Table 3 presents the estimated probabilities of the association between outcome variables (prematurity and LBW) and food security as well as the explanatory variables, employing multivariable logistic regression. The univariate and multivariate logistic regression between premature birth occurrence and household insecurity revealed an insignificant relationship (Table 3). In the adjusted multivariate logistic regression (Table S1), prematurity was significantly associated with maternal education, as mothers with lower education levels were three times more likely to give birth prematurely than mothers with higher educational levels (AOR = 3.23; 95% CI: (1.78–5.84); *p* < 0.001).

In the present study, logistic regression analysis demonstrated a significant association between LBW and household food insecurity after adjusting for confounding factors. It revealed a significant association between LBW and food insecurity, as it showed that LBW was 46% less likely to occur in food-insecure households compared to food-secure households (Table 3).

## 4. Discussion

### 4.1. Low Birth Weight in Food-Secure and Food-Insecure Households

Our findings showed an unexpected result in the association between household food insecurity and LBW. Children experiencing food-insecurity were less likely to have LBW. This observation contrasts with previous findings, which showed that food insecurity was more likely to be associated with LBW [30,31]. Furthermore, previous findings indicated that food insecurity contributes to adverse health outcomes for mothers and children, reflecting maternal nutrition and health [31]. A previous study found that LBW not only denotes a singular health issue but also serves as a sentinel marker for long-term health trajectories. Children born with LBW exhibit increased vulnerability to a spectrum of developmental delays and chronic health conditions, including respiratory illnesses, cardiovascular complications, and higher rates of mortality in infancy and early childhood [32].

Beyond LBW, previous findings revealed that children from food-insecure households had higher risks of suffering with infectious diseases compared to those living in food-secure households [33]. This increased vulnerability could be related to malnutrition, decreased immune systems, and limited access to healthcare, and also the health outcomes that impact children with higher risks in resource-poor settings. For instance, studies show that food insecurity is associated with an increased prevalence of respiratory tract and gastrointestinal infections, as well as other infectious diseases, in young children [34,35]. These health problems not only worsen children’s health, but they can also diminish physical growth, nutrition status, cognitive development, and set the stage for cycles of poverty and poor general health that are prolonged for the next generation. Furthermore, childhood morbidity is significantly associated with both undernutrition and infectious diseases in children under 2 years old.

Our findings highlight the complexity of food insecurity as a social determinant of health and nutrition outcomes among children and mothers. While most studies find that maternal food insecurity is associated with LBW, we found that food insecurity in our cohort was associated with a lower prevalence of LBW. The discrepancies in the findings are currently not understood, but health promotion aimed at improving mothers’ knowledge about fetal growth during pregnancy may have helped to prevent low birth weight [36]. The cash transfers, nutritional interventions, and food aids used in many studies may mitigate the biological impacts and financial burdens that could affect food insecurity during pregnancy [37,38,39]. For instance, cash and food transfers can improve household food availability and, consequently, women’s diet and nutritional status [37,40,41,42].

### 4.2. Prematurity in Food-Secure and Food-Insecure Households

The relationship between food insecurity and prematurity requires further examination. This finding is not consistent with earlier studies, which suggested that household food insecurity is a predictive factor of prematurity [30,43]. However, the present study found that the risk of prematurity in food-insecure households was 1.5 times higher than in food-secure households, inviting a deeper inquiry into the multifaceted determinants of preterm birth. Following prior investigations, prematurity was associated with lower adulthood income [44,45]. Therefore, living in low-income households posed a significantly higher risk of experiencing food insecurity [45].

Our analysis showed that prematurity has a significant relationship with maternal education; that is, mothers with lower education levels were three times more likely to give birth prematurely than mothers with higher education levels, after adjusting for confounding factors. In contrast to previous studies, this study revealed that maternal education may aid in decreasing preterm birth [46]. Higher maternal education is related to better health literacy [47], increased knowledge of healthy diets [48], and greater access to healthcare services [49,50]. This finding analyzed the effect of maternal education on these factors, which may possibly affect the health outcomes of children.

### 4.3. Comparison of Findings in Food-Secure and Food-Insecure Households

The present study reported that lower maternal education corresponds to a heightened chance of food insecurity. This aligns with two findings from a study conducted in Southern Ethiopia, which reported that pregnant women with lower education had a higher risk of being food-insecure compared to the more educated [51,52]. The role of maternal education is to help address household food insecurity by providing the knowledge and skills needed to improve food insecurity and manage resources effectively [53], for instance, by influencing their food choices and child meal plans to enhance adequate nutrition.

Based on a previous study, higher maternal education contributed to employment opportunities and reduced household food insecurity [54]. It indicates that maternal employment contributes to enhancing socioeconomic stability. Our study found that unemployed mothers are two times more likely to be food insecure. Consistent with previous studies in Indonesia, our study indicated that employment status significantly impacts food insecurity, with unemployed mothers more vulnerable to being food insecure [55]. These findings highlight the important role of mothers’ employment status in enhancing household food security, emphasizing the need for policies that promote job opportunities for women to alleviate food insecurity.

As a result, our analysis highlighted that low monthly household income exhibited more than six times higher odds of being food insecure. This aligns with previous findings, which revealed that family income conditions were severely affected by food insecurity during the COVID-19 pandemic [56]. Families with lower income levels and unstable employment status are more prone to food insecurity, amplifying the risks of adverse birth outcomes. Additionally, UNICEF reports pointed out that close to half of the children who experience severe food insecurity are from low-income households [57]. Childhood poverty in early life (in children under five years) could harm and damage child survival, cognitive development, and growth. Moreover, some studies demonstrated that income support significantly reduces the experience of being food-insecure and increases food accessibility [58], particularly by enhancing food purchasing power.

Food insecurity is substantially associated with worse wealth status in a household, with stronger evidence among lower socioeconomic households. Our findings highlighted that households with worse wealth status experience significant food insecurity. Food-insecure households are up to five times more likely to experience food insecurity than their wealthier counterparts. This is consistent with findings from Zimbabwe, which demonstrated that worse wealth status is associated with socioeconomic inequalities. Wealth status was a main predictor of food insecurity and directly related to food accessibility and food purchasing power, affecting adequate nutrition for children [59]. Furthermore, when low wealth status leads to food insecurity, it can further harm health outcomes, particularly in vulnerable risk groups, affecting work capacity and overall well-being, making it difficult for these food-insecure households to improve their wealth status. Likewise, previous research suggested that food insecurity can lead to increased weight and height growth among children [60,61]. This issue indicates that child malnutrition remains alarmingly high in poor households that experience severe food insecurity.

Previous research revealed that underweight children exhibited significantly lower odds of being food secure, demonstrating an inverse relationship between underweight status and food security. This finding aligns with previous findings, which reported that household food insecurity was significantly predictive of malnutrition among children under five, with a particularly significant prediction of being underweight [62]. Similarly, children from food-insecure households have a considerably higher risk of being underweight than children who grew up in food-secure households. Additionally, a previous study found that children in food-insecure households may be nearly five times more likely to be underweight [63]. These findings underscore the urgent need for integrated strategies that aim to address food insecurity in order to improve child nutritional outcomes.

### 4.4. Strengths and Limitations

A significant strength of the study was its comprehensive analysis of the relationship between food insecurity and its predictors, which have relationships with prematurity and LBW. The use of adjusted odds ratios (AOR) strengthens the findings by providing a more precise understanding of the likelihood of adverse health outcomes in food-insecure households after considering confounding factors. Moreover, this study’s analysis identified variables directly associated with prematurity and LBW. Both may have been direct and indirect predictors of food insecurity, which may subsequently exacerbate food insecurity.

The challenge in obtaining representative respondents was one of the limitations of in this study. Even though efforts were made to attempt to make the sample reflect the broader population, certain groups may have limited representation due to a lack of sampling methods, or non-response bias. Additionally, a quantitative method was employed in this study, which identified correlations statistically but may not have identified the experiences behind food insecurity and health issues in children effectively. A qualitative method is suggested to provide future research with more comprehensive results, more complex insights, and deeper connections.

## 5. Conclusions

This study reveals that children born in food-insecure households are at lower risk of low birth weight (LBW) compared to those in food-secure households. Maternal education, maternal employment status, and household income were also found to be closely linked with food insecurity, indicating socioeconomic inequalities that may be crucial in impacting child health outcomes. Further research is required to reveal the causal pathways between food insecurity and birth outcomes and underlying mechanisms, as well as to clarify whether protective community or policy-level factors are involved in both prematurity and birth weight. A comprehensive strategy should be implemented to mitigate food insecurity, prevent its adverse health consequences, and enhance mother and child health. Consequently, government policies must prioritize the empowerment of mothers through education, job opportunities for women, and improvement in access to healthcare services.

## Figures and Tables

**Table 1:** Maternal factors and household characteristics among food-secure and -insecure households in Indonesia (n = 657).

Characteristics	Mean (±SD)	Food Insecure	Food Secure	Total	Chi-Square (*p*-Value)
		n (%)	n (%)	n (%)	
Maternal and household factors					
Mother’s age:	32.79 (±6.21)				0.51
Teen Mothers		0 (0.00)	1 (0.15)	1 (0.15)	
Young Adult Mothers		87 (13.24)	133 (20.24)	220 (33.49)	
Mature Adult Mothers		123 (18.72)	213 (32.42)	336 (51.14)	
Advanced Age Mothers		43 (6.54)	57 (8.68)	100 (15.22)	
Maternal education:					<0.001 *
Low Education		93 (14.16)	90 (13.70)	183 (27.85)	
Medium Education		141 (21.46)	235 (35.77)	376 (57.23)	
High Education		19 (2.89)	79 (12.02)	98 (14.92)	
Mother’s employment status:					<0.001 *
Not employed		166 (25.27)	213 (32.42)	379 (57.69)	
Employed		87 (13.24)	191 (29.07)	278 (42.31)	
Family size:					0.25
Small Family		51 (7.76)	63 (9.59)	114 (17.35)	
Medium Family		170 (25.88)	279 (42.47)	449 (68.34)	
Large Family		32 (4.87)	62 (9.44)	94 (14.31)	
Monthly household income:					<0.001 *
Low income		231 (35.16)	319 (48.55)	550 (83.71)	
Medium income		20 (3.04)	67 (10.20)	87 (13.24)	
High income		2 (0.30)	18 (2.74)	20 (3.04)	
Drinking water:					0.52
Tap water		54 (8.22)	69 (10.50)	123 (18.72)	
Mineral water (in glass/PET bottle)		185 (28.16)	316 (48.10)	501 (76.26)	
Borehole		11 (1.67)	15 (2.28)	26 (3.95)	
Springwater		3 (0.46)	4 (0.61)	7 (1.07)	
House ownership:					0.01 **
Yes		67 (10.20)	146 (22.22)	213 (32.42)	
No		186 (28.31)	258 (39.27)	444 (67.58)	
Car ownership:					<0.001 *
Yes		10 (1.52)	73 (11.11)	83 (12.63)	
No		243 (36.99)	331 (50.38)	574 (87.37)	
Wealth status (self-assessment):					<0.001 *
Rather better off		74 (11.26)	128 (19.48)	202 (30.75)	
Average		164 (24.96)	272 (41.40)	436 (66.36)	
Rather worse off		15 (2.28)	4 (0.61)	19 (2.89)	
Prematurity:	37.33 (±2.40)				0.51
Preterm birth		108 (16.44)	183 (27.85)	291 (44.29)	
Normal birth		145 (22.07)	221 (33.64)	366 (55.71)	
Birth weight:	3.05 (±0.48)				0.07
Low birth weight		17 (2.59)	44 (6.70)	61 (9.28)	
Normal birth weight		236 (35.92)	360 (54.79)	596 (90.72)	

* *p* < 0.001, ** *p* < 0.01.

**Table 2:** Bivariate analysis (Chi-square test) on prematurity and low birth weight (LBW) and its impact factors.

Characteristics	Prematurity			LBW		
	Normaln (%)	Preterm Birthn (%)	*p* Value	No LBWn (%)	LBWn (%)	*p* Value
Child gender:			0.71			0.64
Boy	184 (28.01)	142 (21.61)		294 (44.75)	32 (4.87)	
Girl	182 (27.70)	149 (22.68)		302 (45.97)	29 (4.41)	
Household food insecurity:			0.51			0.07
Food-secure	221 (33.64)	183 (27.85)		360 (54.79)	44 (6.70)	
Food-insecure	108 (22.07)	253 (16.44)		236 (35.92)	17 (2.59)	
Mother’s age:			0.57			0.97
Teen Mothers	0 (0.00)	1 (0.20)		1 (0.20)	0 (0.00)	
Young Adult Mothers	127 (19.30)	93 (14.20)		199 (30.30)	21 (3.20)	
Mature Adult Mothers	182 (27.70)	154 (23.40)		306 (46.60)	30 (4.57)	
Advanced Age Mothers	57 (8.68)	43 (6.50)		90 (13.70)	10 (1.52)	
Maternal education:			0.008			0.11
Low Education	86 (13.09)	97 (14.76)		159 (24.20)	24 (3.65)	
Medium Education	216 (32.88)	160 (24.35)		347 (52.82)	29 (4.41)	
High Education	64 (9.74)	34 (5.18)		90 (13.70)	8 (1.22)	
Mother’s employment status:			0.87			0.09
Not employed	210 (31.96)	169 (25.72)		350 (53.27)	29 (4.41)	
Employed	156 (23.74)	122 (18.57)		246 (37.44)	32 (4.87)	
Family size:			0.76			0.66
Small Family: 3 members	67 (10.20)	47 (7.15)		106 (16.13)	8 (1.22)	
Medium Family: 4–6 members	248 (37.75)	201 (30.59)		405 (61.64)	44 (6.70)	
Large Family: more than 6 members	51 (7.76)	43 (6.54)		85 (12.94)	9 (1.37)	
Monthly household income:			0.09			0.79
Low income	315 (47.95)	235 (35.77)		498 (75.80)	52 (7.91)	
Medium income	39 (5.94)	48 (7.31)		79 (12.02)	8 (1.22)	
High income	12 (1.83)	8 (1.22)		19 (2.89)	1 (0.15)	
Drinking water:			0.12			0.32
Tap water	63 (9.59)	60 (9.13)		113 (17.20)	10 (1.52)	
Mineral water (in glass/PET bottle)	284 (43.23)	217 (33.03)		456 (69.41)	45 (6.85)	
Borehole	17 (2.59)	9 (1.37)		21 (3.20)	5 (0.76)	
Springwater	2 (0.30)	5 (0.76)		6 (0.91)	1 (0.15)	
House ownership:			0.003			0.43
Yes	119 (18.11)	94 (14.31)		196 (29.83)	17 (2.59)	
No	247 (37.6)	197 (29.98)		400 (60.88)	44 (6.7)	
Car ownership:			0.32			0.13
Yes	42 (6.39)	41 (6.24)		79 (12.02)	4 (0.61)	
No	324 (49.32)	250 (38.05)		517 (78.69)	57 (8.68)	
Wealth status:			0.23			0.83
Rather better off	115 (17.50)	87 (13.24)		183 (27.85)	19 (2.89)	
Average	237 (36.07)	199 (30.29)		395 (60.12)	41 (6.24)	
Rather worse off	14 (2.13)	5 (0.76)		18 (2.74)	1 (0.15)	

**Table 3:** Determinant analysis of prematurity, low birth weight, and household food insecurity using logistic regression.

Characteristics	Prematurity		Low Birth Weight (LBW)	
	COR (95%CI)	AOR (95%CI) **	COR (95%CI)	AOR (95%CI) **
Household Food Insecurity:				
Food secure	1	1	1	1
Food insecure	0.90 (0.66–1.23)	0.89 (0.63–1.25)	0.59 (0.33–1.06)	0.54 (0.29–0.99) *

* *p* < 0.05, ** model adjusted for child’s gender, mother’s age, maternal education, mother’s employment status, family size, monthly household income, drinking water, house ownership, car ownership, and wealth status.

## Data Availability

The raw data of this study is available upon request to the authors for the purpose of academic research.

## Supplementary Materials

The following supporting information can be downloaded at: https://www.mdpi.com/article/10.3390/nu17152479/s1, Figure S1: Statistical power analysis of sample test using GPower; Table S1: Adjusted Model Logistic Regression: Prematurity and Low Birth Weight Associated with Food Insecurity.

## Abbreviations

The following abbreviations are used in this manuscript:

LBW	Low birth weight
CI	Confidence interval
AOR	Adjusted odds ratio
SDG	Sustainable development goal
FAO	Food and Agriculture Organization
WHO	World Health Organization
FIES-SM	Food insecurity experience scale survey module
IUGR	Intra-uterine growth restriction
IYCF	Infant and young child feeding
SD	Standard deviation
PET	Polyethylene terephthalate
