# Supplementary material for: Prematurity and Low Birth Weight Among Food-Secure and Food-Insecure Households: A Comparative Study in Surabaya, Indonesia
Source: Nutrients. 2025 Jul 29;17(15):2479. doi: 10.3390/nu17152479 (PMC12348663; doi:10.3390/nu17152479)
Supplement: Supplementary file 1 [file nutrients-17-02479-s001.zip › nutrients-3737699-supplementary.pdf]

Figure S1 Statistical Power Analysis of Sample Test by GPower

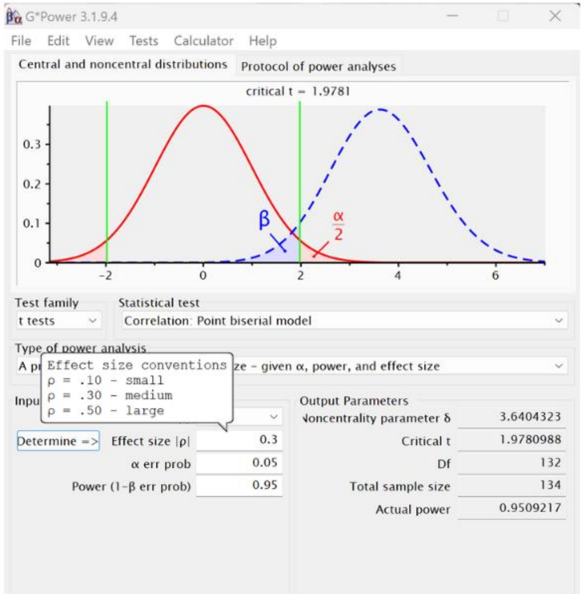

(1) calculating minimum sample

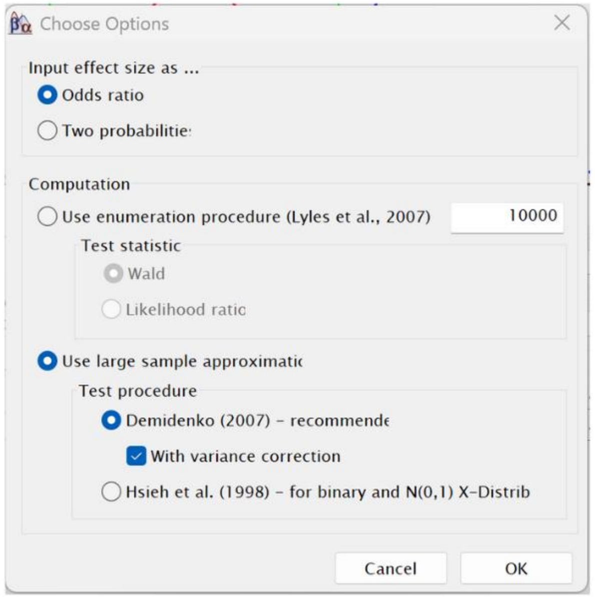

(2) setting up odds ratio

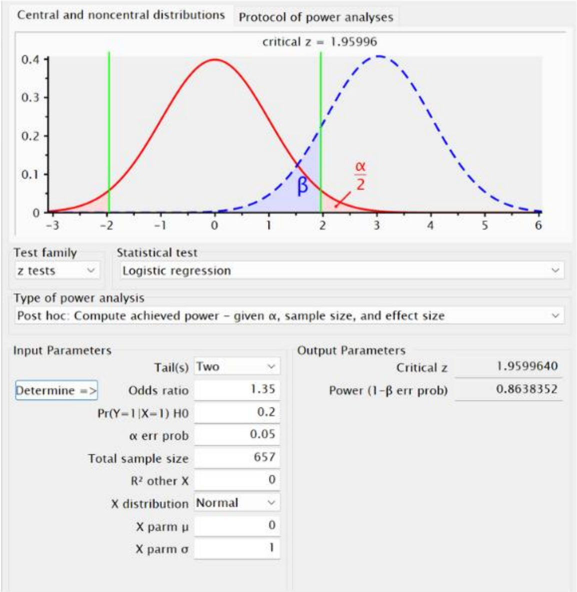

(3) calculating statistical power of sample (n = 657)

Table S1. **Multivariable Logistic Regression: Prematurity and Low Birth Weight Associated with Food Insecurity**

Characteristics	Prematurity		Low Birth Weight (LBW)	
	OR (95% CI)	p value	OR (95% CI)	p value
**Household Food Insecurity**				
Food secure	1		1	
Food insecure	0.88 (0.63 - 1.25)	0.491	0.54 (0.29 - 0.99)	0.046***
**Maternal education**				
Low Education	3.23 (1.78 – 5.84)	0.001*	1.59 (0.62 - 4.12)	0.337
Medium Education	1.90 (1.13 – 3.20)	0.016***	0.83 (0.342 - 1.20)	0.673
High Education	1		1	
**Mothers' employment status**				
Unemployed	0.97 (0.69 – 1.35)	0.844	0.58 (0.33 - 1.02)	0.057
Employed	1		1	
**Family size**				
Small Family: 3 members	0.97 (0.5 – 1.72)	0.919	0.77 (0.27 - 2.16)	0.617
Medium Family: 4-6 members	1.02 (0.64 – 1.63)	0.906	1.26 (0.58 - 2.75)	0.563
Large Family: more than 6 members	1		1	
**Monthly household income**				
Low income	0.93 (0.35 – 2.45)	0.878	1.62 (0.2 - 13.26)	0.654
Medium income	1.73 (0.63 – 4.79)	0.291	1.97 (0.22 - 17.23)	0.541
High income	1		1	
**Car Ownership**				
Yes	1.39 (0.80 – 2.37)	0.241	0.42 (0.13 - 1.31)	0.135
No	1		1	
**House Ownership**				
Yes	1.01 (0.71 – 1.45)	0.938	0.76 (0.401 - 1.42)	0.387
No	1		1	
**Mother's age**				
Teen Mothers	0.98 (0.58 – 1.64)	0.948	1.07 (0.46 - 2.52)	0.873
Young Adult Mothers	1		1	
Mature Adult Mothers	1.16 (0.72 – 1.86)	0.537	0.91 (0.41 - 2.01)	0.818
**Drinking water**				
Tap water	1		1	
Mineral water (in glass/PET bottle)	1.94 (0.78 – 4.84)	0.154	0.45 (0.13 - 1.52)	0.197
Borehole	4.54 (0.71 – 29.22)	0.111	0.65 (0.06 - 7.12)	0.722
Springwater	1.53 (0.65 – 3.62)	0.334	0.49 (0.17 - 1.45)	0.199
**Wealth Status**				
rather better off	1		1	
average	1.11 (0.78 – 1.57)	0.558	0.92 (0.51 - 1.65)	0.771
rather worse off	0.52 (0.17 – 1.55)	0.243	0.59 (0.07 - 4.97)	0.629

*P < 0.001, **P < 0.01, ***p < 0.05
